# Stellate Ganglion Block for Complex Regional Pain Syndrome Treatment After SARS-CoV-2 Vaccine: A Case Report

**DOI:** 10.7759/cureus.38318

**Published:** 2023-04-30

**Authors:** Catarina Nogueira Pinto, Elsa Oliveira, Luís Agualusa

**Affiliations:** 1 Department of Anesthesiology, Unidade Local de Saúde de Matosinhos Hospital Pedro Hispano, Matosinhos, PRT; 2 Department of Pain Medicine, Unidade Local de Saúde de Matosinhos Hospital Pedro Hispano, Matosinhos, PRT

**Keywords:** case report, stroke, stellate ganglion blockade, covid-19 vaccine, complex regional pain syndrome

## Abstract

Complex regional pain syndrome (CRPS) is a poorly understood neuropathic pain syndrome that may have different etiologies. Reports of this syndrome after vaccination are rare. We report a female patient with a medical history of acute stroke of the right carotid artery in the previous four months who developed hyperalgesia, allodynia, edema, and color changes in the upper left member compatible with CRPS one day after SARS-CoV-2 vaccination. A multimodal therapeutic approach was adopted, including a stellate ganglion block, with favorable results, including pain score reduction and increased mobility of the affected member.

## Introduction

Complex regional pain syndrome (CRPS) is a challenging and complex neuropathic pain syndrome. Many causes might be involved in its origin, subdividing CRPS into type I (with no major nerve lesion), type II (with major nerve damage), and CRPS-NOS (not otherwise specified) [1]. The association of CRPS with COVID-19 vaccination is not established. Here, we report a case of CRPS type II four months after a stroke with a possible association with SARS-CoV-2 vaccination.

## Case presentation

A 58-year-old female patient was referred to our pain unit due to a possible CRPS. She had a history of hypertension, dyslipidemia, and a recent acute embolic stroke of an undetermined source of the right carotid artery with striatocapsular and insular ischemia eight months before, complicated with Takotsubo syndrome. In fact, at admission to the emergency room, she presented with left hemiplegia, hemihypoesthesia, asomatognosia, and anosognosia (NIHSS 19). She was submitted to thrombolytic therapy and thrombectomy, scoring NIHSS 10 after the procedure and NIHSS 3 two days after. In the first 24 hours, the patient presented with hypotension corrected by amines, and echocardiography was performed with evidence of apical akinesia. Coronary artery disease was excluded, and Takotsubo syndrome was assumed. The patient was discharged after three days with residual left hemiparesis (grade 4), without limitation on daily activities.

Four months later, pain in the upper left member (especially in the hand and forearm) was developed, associated with edema, warmth, and torsion sensation. The only precipitant factor determined was the administration of the third dose of messenger RNA-based SARS-CoV-2 vaccine (Moderna Spikevax lot 000063A) on the previous day in the left deltoid muscle.

When first evaluated by our team, four months after the beginning of the symptoms, the patient complained of touch sensitivity, allodynia, swelling, and color and temperature changes of the left forearm, wrist, and hand, with an absence of arm pronation or supination, absence of wrist and hand mobilization, and limitation of left shoulder movement. Physical rehabilitation was being performed, and she was previously medicated with gabapentin 100 mg (twice a day), amitriptyline 10 mg, tramadol 37.5 mg with acetaminophen 500 mg (every six hours), prednisolone 40 mg (every 12 hours), and ibuprofen 600 mg as rescue, with no pain relief.

Analgesic therapy was optimized by our team with escalation to gabapentin 300 mg (every eight hours), tramadol 100 mg of prolonged release (every 12 hours), maintenance of amitriptyline 10 mg at night, and supplementation with magnesium and tramadol 37.5 mg with acetaminophen 325 mg as rescue. Non-steroidal anti-inflammatory drugs were suspended, and prednisolone was slowly weaned. Simultaneously, the left cervicothoracic ganglion blockade was proposed. CRPS as a possible adverse effect of the SARS-CoV-2 vaccine was reported by our team at the national adverse events reporting system.

**Figure 1:**
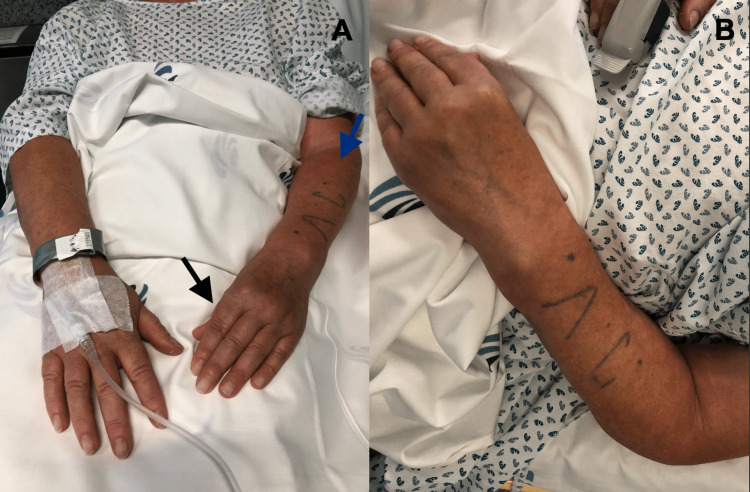
Physical examination findings on the day of the procedure On the day of the procedure, the patient presented with severe wrist rigidity with limitation of mobility adopting left-hand fingers flexion position, particularly thumb flexion (black arrow). Color changes of the left forearm (blue arrow), wrist, and hand are also noted.

The procedure was scheduled after two months. On the day of the procedure, the complaints and physical examination were overlapping (Figure 1).

**Figure 2:**
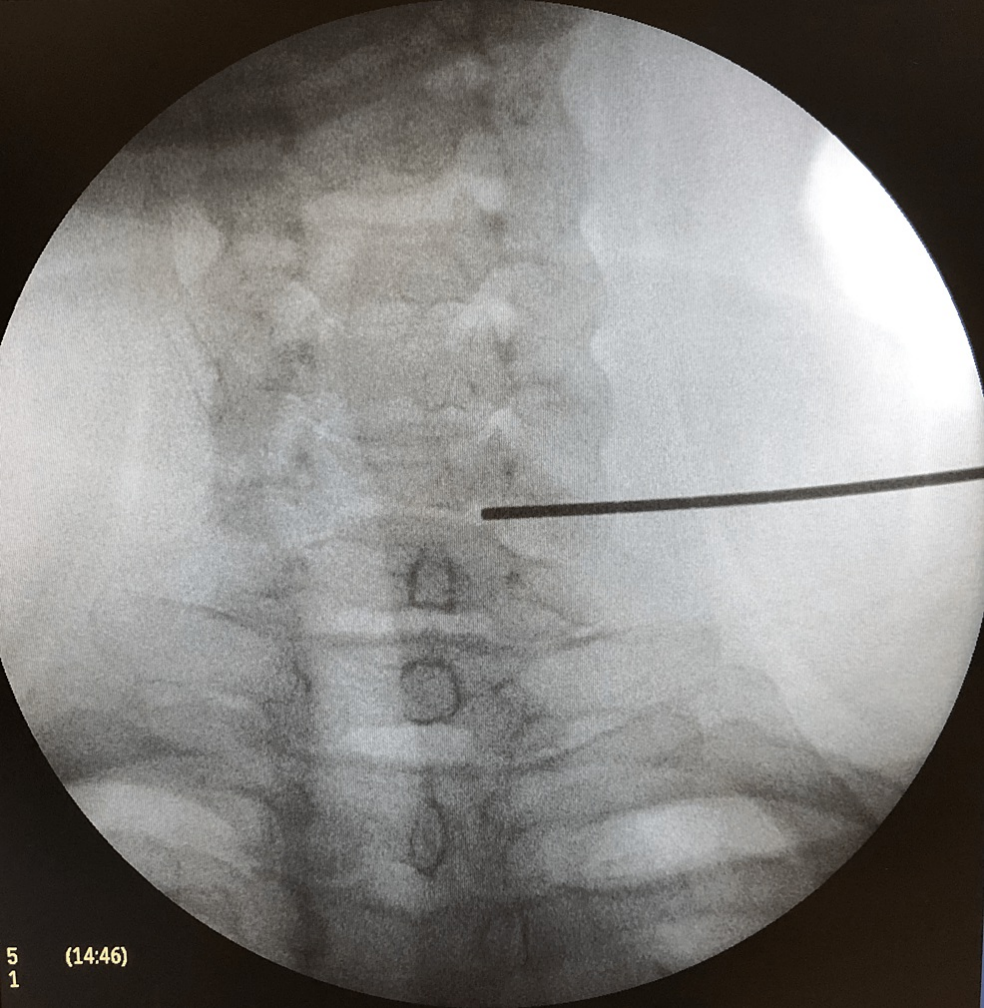
Fluoroscopy image indicating the C6 level

A left cervicothoracic ganglion block was performed, under the American Society of Anesthesiologists standards monitoring, with sedation with a total of 3 mg of midazolam and 0.05 mg of fentanyl. The injection was guided by fluoroscopy and anatomic references at the C6 level (Figure 2).

Locoplex 25 mm x 23G 30° bevel plexus needle (Vygon) was used, and 2 mL of ropivacaine 0.2% and 2 mg of dexamethasone were injected without complications. The procedure had a total duration of 35 minutes.

**Figure 3:**
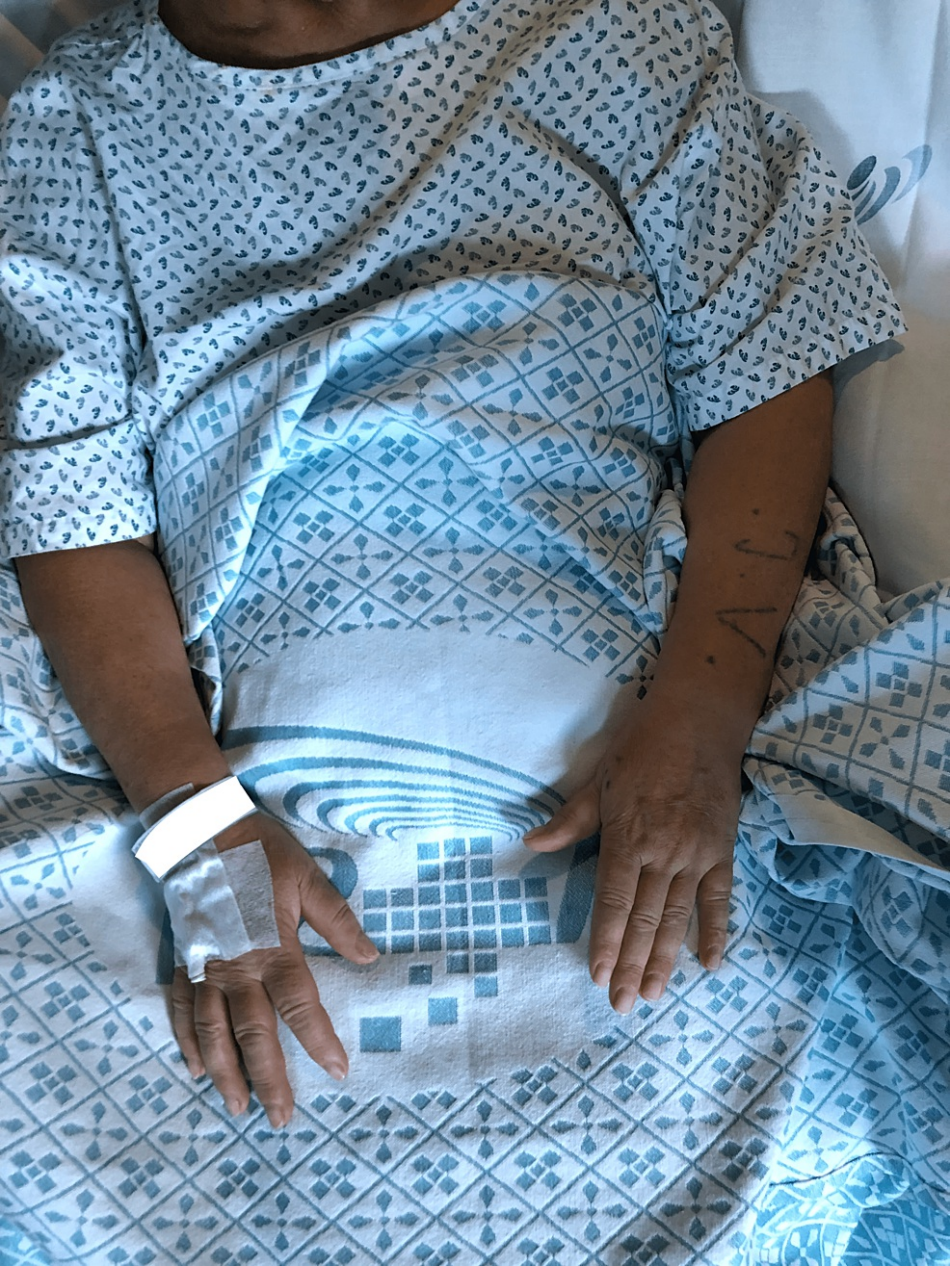
Physical examination findings after the procedure Thirty minutes after the procedure, a significant reduction of left forearm flushing was noted. Simultaneously, modification of the left hand's adopted position is noted, with a reduction of flexion and finger abduction ability.

At the postanesthesia care unit, 30 minutes after the procedure, a significant reduction of flushing (Figure 3) and allodynia of the forearm was noted, and the patient was able to mobilize her hand and wrist, as can be seen in Video 1.

**Video 1:** Left upper member mobility after the procedure After the procedure, a significant reduction of allodynia was noted, and the patient was able to mobilize her left hand and wrist which was not possible immediately before the procedure

Two hours after the procedure. the patient was discharged home. After 24 hours, the patient was contacted for reevaluation, and oral analgesic therapy was weaned (reduction of gabapentin for 300 mg every 12 hours, suspension of tramadol, maintenance of amitriptyline 10 mg and rescue medication), with substantial improvement.

Five days after the procedure, the patient was contacted again and referred for the maintenance of her left-hand mobility and edema regression, with the ability to elevate the left arm to the mouth level. The only complaint was pain from the elbow to the shoulder but with no need for rescue analgesia.

The patient was observed at our pain unit three weeks after the procedure. Physical examination was similar to the previously described, and the patient was very satisfied with the results, with a positive impact on her daily activities, autonomy, and well-being. Simultaneously, the patient was being accompanied by physiotherapists.

## Discussion

Diagnosis and treatment of CRPS are complex and challenging. Both peripheral and central sensitization are involved. It might be described as a type of persistent neuropathic pain syndrome, characterized by moderate to severe pain, hyperalgesia, allodynia, edema, paresthesia, and autonomic and trophic disturbances. The pain intensity is disproportionate to the triggering event and usually doesn’t respect a dermatome territory. The Budapest Criteria make CRPS a diagnosis of exclusion [1].

CRPS has also been found after stroke. The exact pathophysiology remains uncertain, but biomechanical factors and microtrauma to the hemiparetic member may have a significant role in the genesis of CRPS. Of note is the reliance of the glenohumeral joint on the cuff rotator muscles for its stability, predisposing the capsule and extracapsular soft tissue to trauma after muscle atrophy [2]. This might be the reason why shoulder pain was maintained even after the procedure. Inflammation, with elevated levels of interleukin-6, tumor necrosis factor alpha, interleukin-2, hypoxia, with a possible local imbalance of nitrous oxide and endothelin-1 producing acidosis and free radicals, and psychological factors may also be contributors [2].

A case report [3] establishes a possible relationship between COVID-19 and CRPS development, as reported for other viruses, as studies have shown that COVID-19 infection may cause small fiber neuropathy [4]. The incidence of CRPS post-SARS-CoV-2 vaccination is not yet reported [5]. However, one case report and one case series suggest a possible association between the appearance or acute worsening of CRPS symptoms [5-6].

This case report presents a CRPS developed after SARS-CoV-2 vaccination in a patient with a predisposition for this syndrome due to a previous stroke. In fact, the patient didn’t complain of central neuropathic pain after the stroke, and symptoms suggestive of CRPS only appeared one day after COVID-19 vaccination in the same member four months after the stroke.

CRPS presents as a proinflammatory state with dysregulation of the immune system. Autoantibodies have been found in SDRC patients [6]. In fact, activation of alpha 1 adrenergic receptors may cause CRPS pain and dysautonomia symptoms, and activation of beta-2 receptors and M2 anticholinergic receptors may modulate pain and inflammation response [6].

Vaccines work by eliciting an immune response, and, therefore, autoimmune-induced CRPS may be the cause of the appearance of symptoms. Simultaneously, adjuvants used in vaccines to enhance immunological response have been associated with inflammatory conditions [6].

A combination of risk factors including nervous system sensitization, dysregulation of the autonomic system, and inflammatory mediator-related changes contribute to CRPS development [3]. In this case, the patient probably already had some predisposition due to a recent stroke, and the SARS-CoV-2 vaccine was the apparent trigger for CRPS manifestations.

Given the complexity of CRPS, a multimodal and individualized treatment approach is the chosen method, which includes pharmacological, functional, and psychological therapies. An interruption of the sympathetic supply to the affected area is one of the treatment approaches for CRPS since it is hypothesized that, in some cases, the sympathetic nervous system is involved in the pathophysiology [7].

A stellate ganglion block consists of the blockade of the sympathetic ganglia in the lower cervical and upper thoracic region and is a well-recognized approach in CRPS patients, although no particular patient selection or treatment protocol exists.

This case report focuses on the importance of a careful clinical medical history in parallel with chronological events. Although COVID-19 morbidity and mortality are well recognized and vaccination is recommended, its effects are not firmly established. Our case supports others in the literature that suggest a possible relationship between SARS-CoV-2 vaccination and the appearance or recrudescence of CRPS symptoms, as well as the potential contribution to the severity of complaints of a recent stroke.

Given this patient's absence of clinical improvement after pharmacological therapy adjustment, a stellate ganglion block was proposed with significant pain and mobility improvement right after the procedure, which was sustained and had an important impact on daily activities.

## Conclusions

This case report illustrates how a combination of causes of CRPS might simultaneously be present in the same patient. For etiology assessment and treatment guidance, a complete and temporal organized medical history is essential. Here, a possible association between SARS-CoV-2 vaccination and CRPS development was found. Given its complexity, a multidisciplinary approach is needed.
